# Decoding signaling mechanisms: unraveling the targets of guanylate cyclase agonists in cardiovascular and digestive diseases

**DOI:** 10.3389/fphar.2023.1272073

**Published:** 2023-12-20

**Authors:** Qinan Yin, Xingyue Zheng, Yujie Song, Liuyun Wu, Lian Li, Rongsheng Tong, Lizhu Han, Yuan Bian

**Affiliations:** ^1^ Department of Pharmacy, Sichuan Academy of Medical Sciences and Sichuan Provincial People’s Hospital, School of Medicine, University of Electronic Science and Technology of China, Chengdu, China; ^2^ Personalized Drug Therapy Key Laboratory of Sichuan Province, School of Medicine, University of Electronic Science and Technology of China, Chengdu, China

**Keywords:** soluble guanylate cyclase stimulators, soluble guanylate cyclase activators, guanylate cyclase-c agonists, mechanism, signaling pathway

## Abstract

Soluble guanylate cyclase agonists and guanylate cyclase C agonists are two popular drugs for diseases of the cardiovascular system and digestive systems. The common denominator in these conditions is the potential therapeutic target of guanylate cyclase. Thanks to in-depth explorations of their underlying signaling mechanisms, the targets of these drugs are becoming clearer. This review explains the recent research progress regarding potential drugs in this class by introducing representative drugs and current findings on them.

## Graphical Abstract

**Figure:**
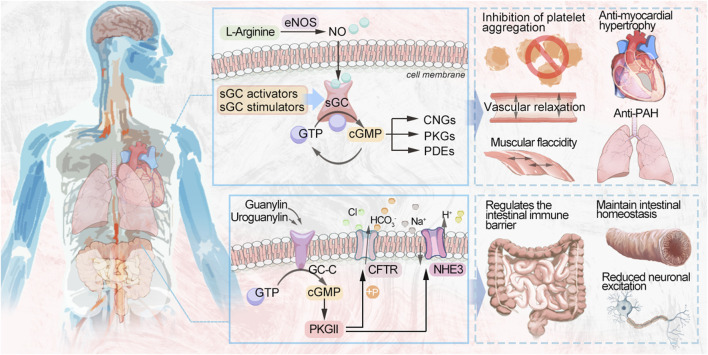
Possible therapeutic indications for sGC agonists and GC-C agonists.

## Introduction

Guanylate cyclase (GC) is a protease that catalyzes the conversion of guanylate triphosphate (GTP) to cyclic guanosine monophosphate (cGMP). Based on the properties of the distributed form of the enzyme, it can be divided into two categories: membrane-bound guanylate cyclase and soluble guanylate cyclase (sGC). Both are widely distributed in the human body, including the lung, brain, kidney, blood vessels, and other tissues (Garbers, 1990). The agonists of membrane-bound guanylate cyclase are peptides (natriuretic peptides A, B, and C), and the agonists of sGC are gaseous mediators (NO and CO) (Grzesk et al., 2023).

sGC is a heterodimer with an α-subunit and β-subunit, of which the latter contains the heme-nitric oxide/oxygen (H-NOX) domain (Argyriou et al., 2021). As a result of its affinity for nitric oxide (NO), this enzyme has also been called an NO-sensitive guanosyl cyclase. NO binding to sGC heme increases GTP cyclase activity, resulting in the production of cGMP, which regulates multiple signaling pathways in cells (Stasch et al., 2011). The cardiovascular, pulmonary, and neurological systems, as well as organs like the kidney, brain, and liver, are highly dependent on NO-sGC-cGMP regulation. Regarding its role in regulation, fibroblasts, cardiomyocytes, platelets, neurons, and immune cells are also affected by cGMP, and it controls fibrosis, the inflammatory response, and neurotransmission (Derbyshire and Marletta, 2009; Grzesk and Nowaczyk, 2021; Nowaczyk et al., 2021).

Guanylate cyclase C (GC-C) is a member of the membrane-bound GC family. It consists of an extracellular domain (ECD) and an intracellular domain, connected by a single strand across the membrane region. In the cell, it synthesizes cGMP and regulates an array of intracellular physiological functions (Hasegawa and Shimonishi, 2005). Reviewing recent progress in research on sGC agonists and GC-C agonists, along with their mechanisms, is the purpose of this paper.

## Soluble guanylate cyclase agonists

sGC is a heterodimeric enzyme with a prosthetic heme group (Archer, 2013). Many physiological functions depend on NO signaling as their primary sensor. sGC binding of NO results in a significant increase in sGC enzyme activity. Additionally, cGMP is produced in a complex with its ligand, NO. The cascades of NO-driven signaling are amplified by sGC (Kang et al., 2019). Only the sGC receptor responds to gaseous NO (Schmidt et al., 2003). cGMPs produced by sGC help regulate the cardiovascular, neuronal, and digestive systems. In pharmacology and therapeutics, improving sGC activity and preventing or reversing inactivation are important goals (Stuehr et al., 2021). There are two forms of sGC in the body: those that respond to NO and those that do not. In brief, sGC contains a heme moiety, which is either ferrous (reduced sGC) or ferric (oxidized sGC). In these forms, the sGC stimulator can only target the reduced, heme-containing sGC, whereas the sGC activator binds to the oxidized or heme-free sGC, resulting in increased production of cGMPs (Dai and Stuehr, 2022). Although sGC activators stimulate heme-containing enzymes independently of NO, NO enhances their activity; even when oxidative stress occurs, cGMP is released by sGC activators (Dai and Stuehr, 2022). These novel pharmacological principles of sGC stimulation and activation appear to have very broad therapeutic potential. Signaling pathways such as NO-sGC-cGMP play an important role in cellular homeostatic maintenance and physiology (Sandner et al., 2021a). The current progress in understanding sGC agonists is summarized in Tables 1, 2.

**TABLE 1:** Current progress on soluble guanylate cyclase stimulators.

Drug	Trial name	Design	Population	Dose	Trial ID	Endpoint	Safety outcome	Stage of development	Conclusion	Reference
Riociguat	PATENT-1	Randomized, Double-blind, Placebo-controlled, Multicenter, Multinational Trial	Patients with PAH	1–2.5 mg tid	NCT00810693	Change in 6MWD	AEs, SAEs and deaths	Completed	Riociguat significantly improved exercise capacity and secondary efficacy end points in patients with PAH	Toxvig et al. (2019)
Riociguat	RESPITE	Open-label, International, Multicenter, Single-arm, Uncontrolled, Phase IIIb Trial	Patients with CTEPH	1–2.5 mg tid	NCT02007629	Change in 6MWD	AEs	Completed	Patients with PAH may benefit from switching from PDE5i to riociguat	Barnikel et al. (2022)
Riociguat	RioSAPH	Double Blind, Placebo Controlled Trial	Patients with SAPH	2.5 mg tid	NCT02625558	Time until clinical worsening	AEs	Unknown	Riociguat was effective in preventing clinical worsening and improving exercise capacity in patients with SAPH.	Baughman et al. (2022)
Riociguat	ISE-IIP	Randomized, Double-blind, Placebo-controlled Phase II Trial	Patients with IIP or PAH	0.5–2.5 mg tid	NCT02138825	Change in PVR at week 26	AEs and SAEs	Terminated	Riociguat should not be used in patients with PH-IIP due to increased serious adverse events and mortality and an unfavorable risk-benefit profile	Nathan et al. (2019)
Riociguat	PATENT-CHILD	Open-label, Individual Trial	Children with PAH	0.5–2.5 mg tid	NCT02562235	Change in 6MWD to Week 16	AEs and SAEs	Active, not recruiting	A suitable riociguat dosing strategy for pediatric patients with PAH have an acceptable safety profile with potential efficacy signals	Garcia et al. (2022)
Riociguat	RISE-SSc	Randomized, Double-Blind, Placebo-Controlled Phase II Trial	Patients with dcSSc	0.5–2.5 mg tid	NCT02283762	Change in mRSS	AEs and SAEs	Completed	Riociguat did not significantly benefit mRSS versus placebo	Khanna et al. (2020)
Vericiguat	SOCRATES-REDUCED	Randomized Parallel-group, Placebo-controlled, Double-blind, Multicenter Phase II Trial	Patients with HF	1.25 mg, 2.5 mg, 5 mg or 10 mg, qd	NCT01951625	Change in NT-proBNP	Changes in LVEF; LVEDV; LVESV	Completed	Vericiguat had no significant effect on NT-proBNP levels in patients with worsening chronic HF and reduced LVEF but was well-tolerated	Gheorghiade et al. (2015)
Vericiguat	VICTORIA	Randomized Parallel-Group, Placebo-Controlled, Double-Blind, Multi-Center Pivotal Phase III Trial	Patients with HF	2.5, 5.0, or 10.0 mg, qd	NCT02861534	Time to Composite Endpoint of Cardiovascular Death or Heart Failure Hospitalization	Symptomatic hypotension and syncope	Completed	Compared to placebo, vericiguat significantly reduced the incidence of the composite endpoint	Armstrong et al. (2020a)
Vericiguat	SOCRATES-PRESERVED	Randomized Parallel-group, Placebo-controlled, Double-blind, Multicenter Dose Finding Phase II Trial	Patients with HFpEF	fixed-dose (1.25 mg or 2.5 mg) and uptitrated to 5 mg or 10 mg, qd	NCT01951638	Change in NT-proBNP and LAV From Baseline	AEs and SAEs	Completed	Vericiguat was well tolerated and did not alter NT-proBNP and LAV at 12 weeks compared to placebo, but was associated with improved quality of life in patients with HFpEF	Pieske et al. (2017)
Vericiguat	VITALITY	Multicenter, randomized, double-blind, placebo-controlled phase IIb trial	Patients with HFpEF	uptitrated to 10 mg or 15 mg, qd	NCT03547583	Change in KCCQ physical limitation score and 6MWD from baseline	TEAEs	Completed	No significant change in KCCQ or 6MWD compared to placebo at 24 weeks	Armstrong et al. (2020b)
Vericiguat	VENICE	Multicenter, Randomized, Placebo-controlled, Double-blind Group Comparison Trial	Patients with CCSs	2.5, 5, or 10 mg, qd	NCT02617550	Measurements of the hemodynamic profile	AEs and SAEs	Completed	The combination of Vericiguat with nitroglycerin administered to patients with CCSs was well tolerated	Boettcher et al. (2022)
Praliciguat	CAPACITY HFpEF	Multicenter, Randomized, Double-blind, Placebo-controlled, Phase 2 Trial	Patients with HFpEF	10, 20, or 40 mg daily	NCT03254485	Change in peak VO2	TEAEs	Completed	These findings do not support the use of praliciguat in patients with HFpEF.	Udelson et al. (2020)
Praliciguat	/	Phase IIA, double-blind, placebo-controlled trial	Patients with 2 diabetes and hypertension	20 mg bid or 40 mg qd	NCT03091920	ABPM and HOMA-IR	TEAEs	Completed	Praliciguat was well tolerated and showed positive trends in metabolic and BP variables	Hanrahan et al. (2020a)
Praliciguat	/	Randomized, Double-Blind, Placebo-Controlled, Phase 2 Trial	Patients with type 2 Diabetes Mellitus combined with Diabetic Nephropathy	20 mg or 40 mg qd	NCT03217591	Change in urine albumin‒creatinine ratio	TEAEs	Completed	Praliciguat treatment did not significantly reduce albuminuria compared with placebo	Hanrahan et al. (2020a)
Olinciguat	/	Multicenter, Randomized, Double-blind, Placebo-controlled, Parallel-group, Single-dose, Phase 2a Study	Patients with Achalasia	Single 5 mg dose	NCT02931565	Change in BFT	TEAEs、SAEs and ADOs	Terminated	/	/
Olinciguat	STRONG SCD	Randomized, Placebo-controlled, Phase 2 Study	Patients with Sickle Cell Disease		NCT03285178	/	TEAEs and SAEs	Completed	/	/

**TABLE 2:** Current progress in soluble guanylate cyclase activators.

Drug	Trial name	Design	Population	Dose	Trial ID	Endpoint	Safety outcome	Stage of development	Conclusion	Reference
Cinaciguat	/	Placebo Controlled, Randomized, Double-blind, Multicenter, Multinational Phase IIb Study	Patients with ADHF	Uptitration from 100–600 μg/g, over maximum 48 h	NCT00559650	Change in PCWP	AEs 、SAEs and TEAEs	Terminated	Cinaciguat unloaded the heart in patients with ADHF. High doses were associated with hypotension	Erdmann et al. (2013)
Cinaciguat	COMPOSE 1	Placebo Controlled, Randomized, Double-Blind, Multicenter, Phase IIb Study	Patients with ADHF	50 μg/h; 100 μg/h or 150 μg/h during 48 h	NCT01065077	Change in PCWP or LOCF	Change in heart rate; SBP; frequency of SAEs and TEAEs	Terminated	It is doubtful that further studies with intravenous cinaciguat would prove beneficial in ADHF patients	Gheorghiade et al. (2012)
Cinaciguat	COMPOSE 2	Placebo Controlled, Randomized, Double-Blind, Multicenter, Phase IIb Study	Patients with ADHF	10 μg/h and 25 μg/h during 48 h	NCT01067859	Change in PCWP or LOCF	Change in heart rate; SBP; frequency of SAEs and TEAEs	Terminated	No statistical analysis was performed	Gheorghiade et al. (2012)
Cinaciguat	COMPOSEE EARLY	placebo controlled, double-blind and randomized study	Patients with ADHF	50 μg/h; 100 μg/h or 150 μg/h during 48 h	NCT01064037	The change in dyspnea assessed using a VAS or LOCF	Change in heart rate; SBP; frequency of SAEs and TEAEs	Terminated	No significant clinical benefit of cinaciguat	Gheorghiade et al. (2012)
Ataciguat	/	Phase Ib Randomized, Placebo-controlled, Double-blinded Study	Patients with Moderate CAVS	50 mg, 100 mg, or 200 mg qd	NCT02049203	The change in blood pressure following the transition from sitting to standing	Number of patients experiencing orthostatic hypotension	Completed	/	/
Ataciguat	SERENEATI	Randomized, Double-blind, Placebo-controlled, Crossover Study	patients with neuropathic pain	200 mg qd	NCT00799656	Change in average daily pain intensity; change in NPSI	Rescue medication intake	Completed	/	/
Ataciguat	ACCELA	Randomized, Double-blind, Placebo-controlled, Parallel Group Trial	Patients with PAD	/	NCT00443287	change in ICD	AEs	Completed	/	/
Ataciguat	CAVS	Phase II Randomized, Placebo-Controlled, Double-Blinded Study	Patients with AVC	200 mg qd	NCT02481258	Changes in Aortic Valve Calcium Levels	/	Completed	/	/
MGV354	/	Randomized, Double-masked, Placebo-Controlled, Safety, Tolerability and Early Efficacy Study	Patients with Ocular Hypertension or Glaucoma	3 μg per eye to 300 μg per eye	NCT02743780	Change in Diurnal IOP	AEs and TEAEs	Completed	Human glaucomatous trabecular meshwork may have levels of oxidized sGC that are too low to benefit from MGV354	Stacy et al. (2018)
Runcaciguat	CONCORD	Randomized, Double-blind, Placebo-controlled, Multicenter Study	Patients with CKD With Diabetes and/or Hypertension	/	NCT04507061	Mean change in UACR	TEAEs; number of subjects with early discontinuations	Completed	/	/
Runcaciguat	NEON-NPDR	Phase 2 Randomized, Placebo-controlled, Double-masked Proof-of-concept Study	Patients with NPDR	Oral dose	NCT04722991	DRSS improvement	TEAEs	Active, not recruiting	/	/
Mosliciguat	ATMOS	Nonrandomized Two Part Multicenter, Open-label, Single Dose Trial	Patients with PAH or CTEPH	up to a maximum dose of 4,000 µg	NCT03754660	reduction in PVR	TEAEs	Completed	/	/
BI-685509	/	Randomized, Double-blind, Placebo-controlled and Parallel Group Trial	Patients with CSPH	/	NCT05161481	Percentage change in HVPG	Decompensation events; CTCAE	Recruiting	/	Reiberger et al. (2023)
BI-685509	/	Randomized, Open-label and Parallel Group Trial	Patients with CSPH		NCT05282121	Percentage change in HVPG	Decompensation events; CTCAE	Recruiting	/	Reiberger et al. (2023)
BI-685509	/	Phase II, Randomized, Placebo-controlled, Double-blind, Parallel Group, Study	Patients with SSc	/	NCT05559580	Rate of decline in FVC	Time to treatment failure	Recruiting	/	/
BI-685509	/	Randomized, Double-blind (Within Dose Groups), Placebo Controlled and Parallel Group Trial	Patients with CKD	/	NCT04736628	Change in UACR	/	Active, not recruiting	/	/
BI-685509	/	Randomized, Double-blind, Placebo-controlled Trial	Patients with DKD	1 mg tid; 3 mg qd; 3 mg tid	NCT03165227	Change in log transformed UACR	AEs 、SAEs	Completed	BI 685509 was generally well tolerated	Cherney et al. (2023)
BI-685509	/	Randomized, Double-blind (Within Dose Groups), Placebo Controlled and Parallel Group Trial	Patients with NPDR	/	NCT04736628	Change in log transformed UACR	/	Active, not recruiting	/	/
BI-685509		Randomized, Double-blind (Within Dose Groups), Placebo-controlled and Parallel Group Trial	Patients with DKD	/	NCT04750577	Change in log transformed UACR	/	Completed	/	/

PCWP, pulmonary capillary wedge pressure; AEs, adverse events; SAEs, serious adverse events; TEAEs, treatment-emergent adverse events; ADHF, acute decompensated heart failure; SBP, systolic blood pressure; VAS, visual analog scale; LOCF, last observation carried forward; CAVS, calcific aortic valve stenosis; NPSI, neuropathic pain symptom inventory; PAD, peripheral arterial disease; ICD, initial claudication distance; AVC, aortic valve calcification; CKD, chronic kidney disease; UACR, urinary albumin-to-creatinine ratio; NPDR, nonproliferative diabetic retinopathy; DRSS, diabetic retinopathy severity scale; PAH, pulmonary arterial hypertension; CTEPH, chronic thromboembolic pulmonary hypertension; CSPH, clinically significant portal hypertension; HVPG, hepatic venous pressure gradient; CTCAE, common terminology criteria for adverse events; SSc, systemic sclerosis; FVC, forced vital capacity; DKD, diabetic kidney disease.

### The NO-sGC-cGMP signaling pathway

**FIGURE 1:**
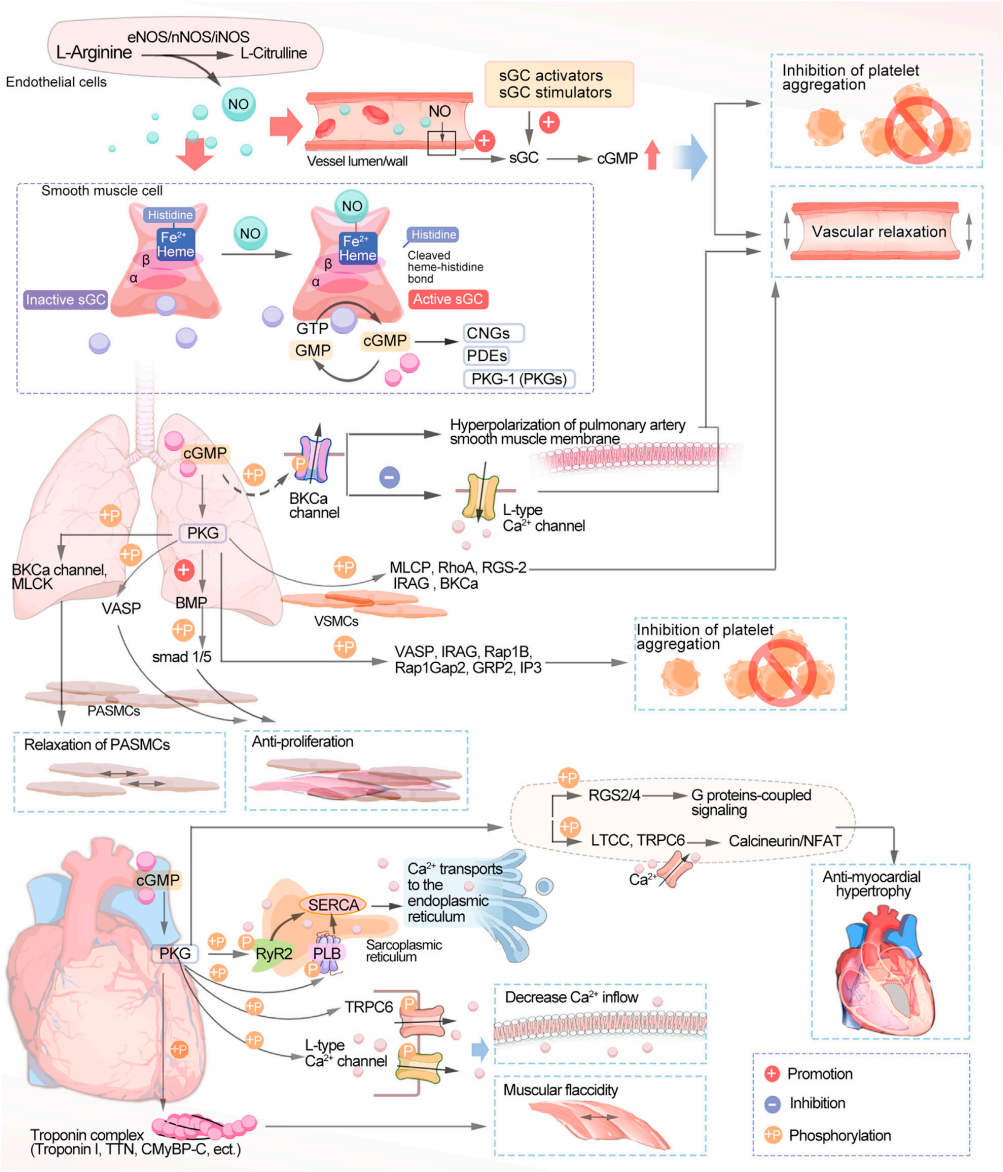
Schematic diagram of the NO-sGC-cGMP signaling pathway. NO, nitric oxide; sGC, soluble guanylate cyclase; cGMP, cyclic guanosine monophosphate; GTP, guanosine triphosphate; CNGs, cyclic nucleotide-gated ion channels; PDEs, phosphodiesterases; PKGs, protein kinases; BKCa, calcium-sensitive potassium channels; MLCK, myosin light chain kinase; VASP, vasodilator-stimulated phosphoprotein; BMP, bone morphogenetic protein; SMAD, small mothers against decapentaplegic; PASMCs, pulmonary artery smooth muscle cells; MLCP, myosin light chain phosphatase; RhoA, Ras homolog family member A; RGS-2, regulator of G-protein signaling 2; Rap1Gap2, Rap1 GTPase-activating protein 2; Rap1b, Ras-related protein 1; IRAG, IP3-induced calcium release; GRP2, guanyl-releasing protein 2; TTN, Titin; CMyBP-C, Cardiac myosin-binding protein-C; SERCA, sarco endoplasmic reticulum Ca^2+^-ATPase; PLB, phospholamban; RyR2, cardiac ryanodine receptor; TRPC6, transient receptor potential canonical channel type 6; RGS, regulator of G protein signaling; LTCCs, L-type calcium channels; NFAT, calcineurin-nuclear factor of activated T cells.

Three different nitric oxide synthases (NOS) are involved in reducing L-arginine to citrulline endogenously (Figure 1): endothelial nitric oxide synthase (eNOS), neuronal nitric oxide synthase (nNOS), and induced nitric oxide synthase (iNOS) (Sandner et al., 2018). During vascular NO-sGC-cGMP signaling, L-arginine is converted to NO in the endothelial monolayer by endothelial nitric oxide synthase (eNOS) and diffuses into the vascular lumen and the vessel wall, thereby activating sGC. Heme-dependent sGC stimulators and nonheme-dependent sGC activators increase cellular cGMP concentrations by directly activating sGC, leading to vascular relaxation and inhibiting platelet aggregation (Evora et al., 2012). NO produced through endothelial cells plays an important biological role. cGMP is synthesized by NO by binding to the active heme-containing sGC in vascular smooth muscle cells. Smooth muscle sGCs are NO signaling targets. The binding of NO to sGC leads to the conversion of GTP to cGMP. The resulting cGMP is hydrolyzed after binding to one of three types of cGMP effector proteins, including gated cation channels, the protein kinases (PKGs) that are dependent on cGMP, and the phosphodiesterases (PDEs) that are regulated by it (Feil et al., 2022).

Disruption of the NO-sGC-cGMP signaling pathway is central to the pathogenesis of pulmonary arterial hypertension (PAH) and chronic thromboembolic pulmonary hypertension (CTEPH), in which endothelial dysfunction leads to impaired NO synthesis. The progression of PAH and CTEPH is also associated with low NO production. The sGC stimulators have a dual role in that they directly stimulate the native form of the enzyme, making it more sensitive to endogenous NO and increasing sGC activity regardless of NO and cGMP levels, leading to an increase in cGMP. (Benza et al., 2019). The vasodilation effect of cGMP in the pulmonary circulation is mediated by a variety of subcellular mechanisms, one of which is the activation of cGMP-dependent protein kinase, phosphorylating calcium-sensitive potassium channels (BKCa), leading to potential hyperpolarization of the pulmonary artery smooth muscle membrane and inhibiting calcium inflow through L-type Ca^2+^ channels (LTCCs). sGC is a redox-sensitive enzyme that is activated by hydrogen peroxide, causing pulmonary artery blood vessels to dilate. However, in cases of excessive oxidative stress, as occurs in disease states, reactive oxygen species or nitrosylation can change the oxidation state of sGC from normal reduced heme iron (Fe^2+^) to oxidized heme (Fe^3+^), making it less active and less responsive to NO. The oxidized sGC then loses its heme portion, after which it will eventually be degraded by the proteasome. The heme-free form of sGC is the target of sGC activators (Dasgupta et al., 2015). In the pulmonary circulation, NO acts as an endogenous pulmonary vasodilator and that synthesized by the action of eNOS on L-arginine in pulmonary vascular endothelial cells and then diffuses to adjacent vascular smooth muscle cells (VSMCs) to activate sGC (Triposkiadis et al., 2022). In VSMCs, MLCP, RhoA, RGS-2, IRAG, and BKCa are phosphorylated by PKG to promote vasodilation (Klinger and Kadowitz, 2017; Sadek et al., 2020). Many proteins are changed in their phosphorylation state when endogenous PKG is activated, including VASP and IRAG, Rap1B and Rap1Gap2, GRP2, and IP3 receptors, among others, which inhibit platelets as well (Gambaryan, 2022). Several studies have suggested vasoconstriction/vasodilation and proliferation/anti-proliferation alterations as possible pathogenic mechanisms of PAH (Biswas et al., 2020). PKG acts in pulmonary artery smooth muscle cells (PASMCs) by several mechanisms. PKG phosphorylates the BKCa channel and MLCK, leading to the relaxation of PASMCs (Christou and Khalil, 2022; Barenco-Marins et al., 2023). Vasodilator-stimulated phosphoprotein (VASP) is an actin-binding protein, and phosphorylation of VASP by PKG inhibits PASMC proliferation (Chen et al., 2004). Bone morphogenetic protein (BMP) is a signaling molecule belonging to the transforming growth factor-β (TGF-β) superfamily, which plays an important role in regulating cell proliferation, differentiation, and apoptosis. Recent studies have found that PKGI enhances the phosphorylation of the downstream signal small mothers against decapentaplegic (SMAD) 1/5 by BMP, promotes the antiproliferative and pro-differentiation effects of BMP, and keeps PASMCs in a proliferation-inhibited state (Watanabe, 2018).

The NO-sGC-cGMP signaling pathway maintains the normal function of the cardiovascular system in healthy individuals, and sGC activity falls as heart failure with reduced ejection fraction (HFrEF) progresses due to endothelial dysfunction and oxidative stress. sGC stimulation leads to increased cGMP synthesis, which can inhibit myocardial fibrosis, reduce vascular wall hardness, and induce vasodilation (Belenkov and Kozhevnikova, 2023). A lack of sGC stimulation and a decrease in cGMP production are associated with heart failure (HF) and decreased NO bioavailability. Elevated levels of plasma inflammatory cytokines, including TNF-α and IL-6, in patients with HF are associated with endothelial dysfunction with low NO-sGC-cGMP signaling in the heart and blood vessels (Numata and Takimoto, 2022). Impairment of the NO-sGC-cGMP pathway in HF, a key second messenger pathway mediating vascular and cardiac dilation, in patients with dysfunction of the endothelium, myocardium, and blood vessels may play a role in the progression of cardiovascular disease (CVD). Notably, both HFrEF and heart failure with preserved ejection fraction (HFpEF) patients have cGMP deficiency. There are also cases where oxidative stress results in heart muscle cell loss, collagen replacement, and fibrosis due to autophagy, apoptosis, or necrosis (Hulot et al., 2021). Disruption of the NO-sGC-cGMP pathway results in the narrowing of blood vessels, clumping of platelets, inflammation, scarring, and notably, maladaptive enlargement of the heart. Hence, NO-sGC-cGMP pathway restoration is a promising pharmacological target for HF treatment (Rudebusch et al., 2022). In the heart, PKG phosphorylates phospholamban (PLB) (Eggermont et al., 1988) and RyR2 (Xiao et al., 2006), thus activating sarcoplasmic reticulum Ca^2+^ ATPase (SERCA) to promote the transport of calcium ions to the endoplasmic reticulum. In addition, PKG can phosphorylate numerous membrane channels, including L-type calcium channels (Yang et al., 2007) and transient receptor potential typical channel type 6. This ultimately reduces the inflow of extracellular calcium. Troponin I (Tsai and Kass, 2009), titin (Kruger et al., 2009), and CMyBP-C (Thoonen et al., 2015) act as structural proteins that regulate contraction of the myocardium, leading to myocardial relaxation through phosphorylation of PKG. PDE5 breaks down cGMP in cardiomyocytes into GTP, which is eventually recycled (Mihalek et al., 2022). Its activity counteracts vascular constriction and helps maintain vital organs, the highest expression of sGC being found in perfusion cardiomyocytes. cGMP produced by sGC causes ventricular diastole and decreased contractility (Hulot et al., 2021). In anti-myocardial hypertrophy, regulators of G-protein signaling (RGS), namely, RGS2 and RGS4, have a central role, leading to cGMP-mediated anti-myocardial hypertrophic effects by inactivating G-protein-coupled signaling, as RGS2 and RGS4 are targets of PKG (Klaiber et al., 2010). The Ca^2+^-dependent mechanism of calcitonin/nFAT hypertrophy is further inhibited by the phosphorylation of the LTCC and transient receptor potential canonical channel type 6 (TRPC6) channels by cGMP/PKG (Kinoshita et al., 2010), and these mechanisms may be related to cGMP-mediated antihypertrophy, antifibrosis and alleviation of cardiac dysfunction (Sandner and Stasch, 2017).

Since the NO-cGMP signaling cascade is active in many tissues *in vivo*, the pathway is currently being explored for other roles in conditions other than cardiovascular disease, such as chronic kidney disease, fibrotic disease, neuroprotection, and dementia (Sandner, 2018). TGF-β signaling plays an important role in cellular fibrosis, and several preclinical studies have demonstrated that inhibition of TGF-β signaling exerts a potent antifibrotic effect in different organs in a variety of animal models (Distler et al., 2019; Englert et al., 2023). PKG inhibits the phosphorylation of extracellular signal-regulated kinase (ERK) by TGF-β signaling, suppresses ERK signaling, and prevents its translocation to the nucleus, blocking TGF-β-mediated extracellular matrix (ECM) generation, fibroblast differentiation to myofibroblasts, and cell proliferation (Hu et al., 2017; Sandner and Stasch, 2017).

Furthermore, cGMP modulates renal blood flow, renin secretion, glomerular function, and tubular exchange processes through its direct effects on cGMP signaling cascades. It is possible to develop renal diseases such as chronic kidney disease (CKD) when NO-sGC-cGMP signaling is downregulated. As a result, therapeutic strategies that maintain or increase cGMP activity may be effective against progressive kidney disease (Krishnan et al., 2018).

#### Riociguat

Currently, riociguat is the only FDA-approved sGC stimulator for treating PAH and CTEPH. It is an orally administered drug that can be rapidly absorbed, with a bioavailability of 94.3%. Riociguat’s half-life varies significantly from individual to individual, at approximately 12 h for PAH/CTEPH patients and 7 h for healthy people. Riociguat promotes cGMP synthesis. It has shown good efficacy in clinical trials and is well tolerated. Riociguat has a valuable place in the treatment of pulmonary hypertension (Kenny et al., 2022).

#### Riociguat in PAH

The IPATENT-1 trial (NCT00810693) found that riociguat significantly improved the 6-min walking distance of PAH patients by 36 m (m) over placebo. The PHIRST-1 trial (NCT00125918) and the SUPER-1 trial (NCT00644605) trials showed that riociguat had similar results to those of tadalafil (mean difference of 33 m) and sildenafil (mean difference of 50 m), which are both rivals of this product. Pharmacokinetics (PK) and adverse events (AEs) were also similar. Riociguat was used in the RESPITE study (NCT02007629) to treat PAH patients who failed to respond adequately to either tadalafil or sildenafil. Based on these findings, riociguat may outperform PDE-5 inhibitors in efficacy (Toxvig et al., 2019). Riociguat has not shown any new safety signals since the FDA approved it in 2013, according to an evidence-based review of its safety and tolerability. The overall incidence of AEs was 93%–96%. The most common AE types are nasopharyngitis and peripheral edema. The most common severe AEs (SAEs) include syncope, right ventricular (RV) failure, hypotension, and hemoptysis. Although common side effects are reported, they are tolerated in 87%–92% of people (Donaldson et al., 2020). It is also well tolerated in older patients (Barnikel et al., 2022). Study SE-IIP (NCT02138825) of idiopathic interstitial pneumonia with pulmonary hypertension (PH) was terminated early, as patients taking riociguat experienced a higher occurrence of SAEs and mortality, as well as no efficacy signal. In the main study, 11 patients died (8 in the riociguat group and 3 in the placebo group); riociguat was associated with more SAEs among PH-IIP patients, as well as adverse risk/benefit profiles. Therefore, patients with PH-IIP should avoid using riociguat (Nathan et al., 2019). This meta-analysis encompassed the incorporation of eight randomized controlled trials with 1,606 participants of riociguat’s effects on all types of PH. For PAH and CTEPH patients, riociguat treatment significantly extended the 6MWD compared with placebo and decreased N-terminal pro-B-type natriuretic peptide (NT-proBNP), mean pulmonary artery pressure (PAP), pulmonary vascular resistance (PVR), and right atrial pressure (RAP). The cardiac index (CI) increased, cardiac output increased, and adverse events and clinical exacerbations decreased. A significant difference in efficacy outcomes and safety outcomes was not observed in other types of PH. Patients with PAH and CTEPH benefit from riociguat treatment, but patients with other types of PH only see partial hemodynamic improvements (Wang et al., 2021). The haemoDYNAMIC trial (NCT02744339) was a placebo-controlled, randomized, double-blind clinical trial that enrolled 114 patients with PH combined with HFpEF, who were randomly assigned to take riociguat or placebo. At 26 weeks, the riociguat group was significantly better in the primary efficacy measure, resting cardiac output, as determined through a right cardiac catheter (Dachs et al., 2022). In a multicenter, phase III, open-label, randomized controlled trial (NCT02634203), at week 26, riociguat therapy resulted in a more significant decrease in pulmonary vascular resistance than balloon pulmonary angioplasty (BPA) in 53 inoperable CTEPH patients, while BPA was performed on 52 patients. Treatment-related SAEs occurred more frequently in the BPA group: 22 of 52 (42%) of them experienced treatment-related SAEs vs. 5 of 53 (9%) in the riociguat group. Out of the 52 patients in the BPA group, 18 (35%) experienced lung injury, while 2 out of 53 patients (4%) reported severe hypotension leading to syncope. Deaths related to treatment were not reported (Jais et al., 2022). For patients requiring combination therapy, one review mentioned the possibility of pretreatment with riociguat plus an endothelin receptor antagonist in patients with PAH at high risk of death after 1 year (Rahaghi et al., 2023). A trial was conducted to test the combination of riociguat and ambrisentan in patients suffering from functional grade III PAH using a prospective, single-arm, open-label approach (NCT02634203) (Weatherald et al., 2022). In patients with sarcoidosis-associated pulmonary hypertension (SAPH), a double-blind placebo-controlled trial compared riociguat with placebo and examined the outcome of prolonged clinical worsening events (NCT02625558). Sixteen patients were randomized to riociguat (*n* = 8) and placebo (*n* = 8). By log-rank analysis, patients treated with riociguat stayed in the study significantly longer, their 6MWD scores tending to increase. At 1 year, riociguat was effective in preventing deterioration and in improving motor capacity in patients with clinical SAPH (Baughman et al., 2022). Riociguat is currently approved for use in adults only. It was tested for use in children in a multicenter, single-trial, 24-week, open-label phase 3 study, PATENT-CHILD (NCT02562235). The PK and safety of oral riociguat in pediatric patients with PAH were evaluated in World Health Organization Functional Classification (WHO-FC) Grade I-III patients aged 6–17 years who were being treated with stable endothelin receptor antagonists and/or prostacyclin analogs. They were given 0.5–2.5 mg of riociguat three times a day. A total of twenty-four individuals, with an average age of 12.8 years, were enrolled. Eighteen of these were WHO-FC II. Twenty patients (83%) reported primarily mild or moderate AEs. SAEs occurred in 4 cases (17%). All problems were resolved by the end of the study, and two out of the total (8%) were believed to be associated with the experimental medication. There were 3 cases of hypotension and 1 case of hemoptysis (all mild/moderate intensity). These children had similar blood concentrations of riociguat to those published in adult patients. From baseline to week 24, the mean ± standard deviation of the 6-min walking distance of patients increased by 23 ± 69 m (*n* = 19), and the mean NT-proBNP decreased by −66 ± 585 pg/mL (*n* = 14). The WHO-FC of the patients did not change. A clinical worsening event occurred in two patients after they were hospitalized with right heart failure. Riociguat is a safe and effective treatment for pediatric PAH, based on the PK results. The data suggest that riociguat has an acceptable safety profile and a potential efficacy signal in a pediatric cohort (Garcia et al., 2022).

#### Anti-cardiac-remodeling effects of riociguat

To test the hypothesis that sGC stimulation with riociguat can prevent pathological cardiac remodeling and HF caused by chronic pressure overload, an animal model of C57BL/6N mouse HF was established. After 3 weeks of transverse aortic constriction (TAC) treatment, the animals were randomized to receive riociguat or its solvent (Sol). After 5 weeks of treatment, the left ventricular rejection fraction (LVEF) improved (TAC + Rio 43.4% ± 6.0%, TAC + Sol 20.9% ± 4.3%; *p* < 0.001), and the left ventricular mass to body weight (LVM/BW) ratio, myocardial fibrosis, and the myocyte cross-sectional area decreased (5.5 ± 0.4 mg/g vs 7.7 ± 0.7 mg/g; *p* < 0.001). RNA sequencing indicated that riociguat administration reduced the expression of myocardial remodeling genes (e.g., Nppa, Nppb, Myh7, collagen genes) and downregulated signaling pathways associated with hypertrophic cardiomyopathy and heart failure. Muscle and fibroblast cultures showed a reversal of pathological stress responses when riociguat was administered (Rudebusch et al., 2022). Another bioinformatic analysis of C57BL/6N mice subjected to TAC showed that riociguat administration improved the expression of markers of pathways, metabolism, and energy production related to cardiovascular disease induced by TAC. Changes in the levels of myosin heavy chain 7 (MYH7), cardiac phosphoprotein (PLN), and ankyrin repeat domain-containing protein 1 (ANKRD1) were reversed. Riociguat also attenuates TAC-induced changes in left ventricular microRNA levels. This suggests that riociguat has beneficial effects on cardiac structure and function during stress overload, supporting the potential use of riociguat as a novel treatment for HF (Benkner et al., 2022).

#### Riociguat in diffuse cutaneous systemic sclerosis (dcSSc) and peripheral arterial disease

The effectiveness and safety of riociguat in patients at high risk of progressing skin fibrosis in early dcSSc were evaluated in a randomized, double-blind, placebo-controlled phase 1b trial (NCT02283762), an 18-month study of 60 participants with a modified Rodman skin score (mRSS) of 10–22 units who received riociguat orally three times a day for 18 months (*n* = 60). At week 52, the change in mRSS units from baseline was −2.09 ± 5.66 (*n* = 57) in the riociguat group and −0.77 ± 8.24 (*n* = 52) in the placebo group (mean least squares difference was −2.34 (95% CI: −4.99–0.30) (*p* = 0.08). Among patients with interstitial lung disease, forced lung capacity decreased by 2.7% in the riociguat group and 7.6% in the placebo group. In the riociguat group, 41.3% (19 out of 46) of patients showed an improvement of 250% in their Raynaud scores at week 14, compared to 26.0% (13 out of 50) in the placebo group. In the safety assessment, no new signals of inflammation or treatment-related death were identified. Riociguat had no significant benefit on mRSS units. A secondary analysis and exploratory analysis revealed potential efficacy signals worth testing in further trials. Riociguat was well tolerated (Khanna et al., 2020).

Researchers have studied new blood vessel formation in mice with limb ischemia following the administration of riociguat. A 28-day treatment with 3 mg/kg/d riociguat was given intragastrically to C57BL/6 mice. Induction of posterior limb ischemia was achieved by surgically removing the femoral artery after 2 weeks of treatment. *In vitro* matrix tests showed that riociguat stimulated tubule formation in human umbilical vein endothelial cells (HUVECs) in a dose-dependent manner. Cell migration (assessed by scratch test) indices were also increased in HUVECs treated with riociguat compared to controls. At the cellular level, administration of riociguat resulted in swift initiation of the p44/p42 mitogen-activated protein kinase (MAPK) cascade in HUVECs. HUVECs treated with riociguat exhibited a reduced response to PKG inhibition. During ischemia, riociguat therapy improves blood flow recovery (as measured by laser Doppler imaging) and increases capillary density in ischemic muscle (as measured by CD31 immunostaining). The clinical results have shown that these indices reduce movement disorders and ischemic injuries significantly. The number of bone marrow-derived proangiogenic cells (PACs) was also increased by 94% when mice were treated with riociguat. Riociguat treatment was associated with significant improvements in PAC function, including migration ability, adherence to endothelial monolayers, and integration into the endodermal tubular network. GC stimulants can promote angiogenesis and improve neovascularization after ischemia, whose mechanism involves PKG-dependent p44/p42 MAPK activation, an improvement in PAC function, and an increase in their quantity. In patients with severe atherosclerosis, sGC stimulation may offer a novel approach to reducing tissue ischemia (Dhahri et al., 2023).

#### Vericiguat

Vericiguat was the first sGC stimulator to be marketed as of 2021, for patients with symptomatic chronic heart failure accompanied by an ejection fraction of 45%, to reduce the risk of cardiovascular death and heart failure hospitalization after heart failure hospitalization or the need for outpatient IV diuretics (Markham and Duggan, 2021). Vericiguat works synergistically with endogenous NO and enhances the affinity of sGC for low levels of NO (Singh et al., 2017). Therefore, vericiguat therapy is expected to restore the activity of an impaired NO-sGC-cGMP pathway, resulting in a variety of pharmacological effects, including improved cardiac and vascular function and reduced levels of profibrotic and inflammatory pathway markers. By increasing cGMP levels, vericiguat can also cause vascular relaxation and enhance the control of vascular tone and myocardial dysfunction (Hulot et al., 2021).

The average steady-state vericiguat distribution volume in healthy subjects is approximately 44 L, with a high bioavailability of approximately 93% when taken with food at a dose of 10 mg. Its serum albumin binding rate is approximately 98%, and its clearance rate is 1.6%. L/h. It takes 30 h for vericiguat to reach half-life in patients with HF. Ninety-five percent of the vericiguat dose is metabolized primarily by glucosylation of UGT1A1 (minor) and UGT1A9 (major), resulting in inactive N-glucuronic acid metabolites. CYP450-mediated metabolism is a minor clearance pathway, accounting for only 5% of the total clearance capacity. Fifty-three percent of the metabolized drug is excreted in urine, and 45% is excreted in feces (Campbell et al., 2022; Chiles and Al-Horani, 2022). Vericiguat, at doses up to 10.0 mg QD for 7 days, is generally well tolerated in healthy men in Europe, China, and Japan. Oral vericiguat 15.0 mg was not well tolerated, and drug-related treatment-emergent adverse events (TEAEs) were predominantly neurologic disorders such as headache and postural dizziness, which may be related to the mode of action of vericiguat (namely, vasodilation) (Boettcher et al., 2021).

#### Vericiguat in HFrEF

Preliminary evidence regarding vericiguat in the context of HFrEF was derived from two clinical trials, namely, the phase II study SOCRATES-REDUCED and the phase III study VICTORIA (Vannuccini et al., 2022). Four hundred fifty-six patients with clinical stability with an LVEF less than 45% within 4 weeks of worsening chronic heart failure, defined as congestion symptoms and elevated natriuretic peptide levels requiring inpatient or outpatient intravenous diuretics, were randomly assigned to an arm of the SOCRATES-REDUCED trial (NCT01951625). Overall, 351 patients (77.0%) completed investigational drug therapy with 12 weeks of effective NT-proBNP levels without major protocol bias, meeting the criteria for primary endpoint evaluation. In the preliminary analysis, the AE rates were 77.2% and 71.4% in the placebo and 10 mg vericiguat groups, respectively. The effects of vericiguat on NT-proBNP levels at 12 weeks in patients with chronic heart failure exacerbations and reduced LVEF were not significant compared with placebo, but the vericiguat was well tolerated. This drug had a dose‒response relationship, indicating that further clinical trials will be needed to determine the effectiveness of the drug in treating worsening chronic heart failure patients (Gheorghiade et al., 2015). In the VICTORIA III trial (NCT02861534), 5,050 patients were diagnosed with chronic heart failure (class II, III, or IV according to the New York Heart Association) with an ejection fraction of less than 45%. They were given vericiguat (10 mg once daily, target dose) or placebo (no treatment), along with guideline-based medication. The combined result of cardiovascular death and the first hospitalization for heart failure was the primary outcome. At a median of 10.8 months, 897 of 2,526 patients (35.5%) in the vericiguat group had had a primary outcome event, compared with 972 of 2,524 patients (38.5%) in the placebo group (hazard ratio, 0.90; 95% CI, 0.82–0.98; *p* = 0.02). A total of 691 (27.4%) patients were hospitalized for heart failure in the vericiguat group *versus* 747 (29.6%) patients in the placebo group (hazard ratio 0.90; 95% CI, 0.81–1.00). There were 414 (16.4%) deaths from cardiovascular causes in the vericiguat group and 441 (17.5%) deaths from cardiovascular causes in the placebo group (hazard ratio, 0.93; 95% CI, 0.81–1.06). A total of 957 (37.9%) patients died from any cause or were hospitalized for heart failure in the vericiguat group *versus* 1,032 (40.9%) patients in the placebo group (hazard ratio: 0.90; 95% CI, 0.83–0.98; *p* = 0.02). Symptomatic hypotension occurred in 9.1% and 7.9% of patients in the vericiguat and placebo groups (*p* = 0.12), respectively, and syncope occurred in 4.0% and 3.5% of patients in the vericiguat and placebo groups (*p* = 0.30), respectively. In conclusion, among high-risk patients with heart failure, treatment with vericiguat led to a lower incidence of death from cardiovascular causes or hospitalization for heart failure than placebo (Armstrong et al., 2020a).

#### Vericiguat in HFpEF

Socrates-preserved (NCT01951638) was a 12-week, double-blind, placebo-controlled, phase 2b trial of 447 patients with deteriorating symptoms of chronic hypertension and an LVEF ≥45% (Pieske et al., 2017). Patients were randomized to receive placebo or vericiguat once daily (1.25, 2.5, 2.5–5.0, or 2.5–10.0 mg). Patients tolerated vericiguat well (AEs: vericiguat 10 mg arm, 69.8%; placebo, 73.1%), but the two groups had similar changes from baseline in NT-proBNP (one-sided *p* = 0.90, two-sided *p* = 0.20) and left atrial volume (one-sided *p* = 0.81, two-sided *p* = 0.37) at 12 weeks. A larger proportion of patients treated with 10 mg vericiguat achieved clinically meaningful improvements in the Kansas City Cardiomyopathy Questionnaire Clinical Summary Score (KCCQ-CSS) and the 5-dimensional EuroQol questionnaire (EQ-5D). Given the encouraging results in terms of quality of life, there is a need for further studies on the impact of vericiguat in patients with HF. The VITALITY study (NCT03547583) was designed to understand the efficacy and safety of vericiguat on quality of life and exercise tolerance in patients with HF and HFpEF (Armstrong et al., 2020b). A total of 789 patients had chronic HF, EF ≥ 45%, New York Heart Association functional class II or III, decompensation in the last 6 months (no hospitalization due to HF or need for diuretics for intravenous treatment of HF), and elevated natriuretic peptides. Patients were randomized to receive up to 15 mg (n = 264), 10 mg (n = 263) or placebo (n = 262). No significant differences were found between groups in the physical limitation score of the KCCQ or the 6MWD after 24 weeks of treatment. The different outcomes between SOCRATES-PRESERVED and VITALITY in terms of improving quality of life in patients with HFpEF are of interest and require further investigation.

#### Vericiguat in chronic coronary syndromes

Vericiguat plus nitroglycerin was compared to nitroglycerin alone for safety, tolerability, and pharmacodynamic effects in patients with chronic coronary syndromes (CCSs). The VENICE (NCT02617550) randomized, double-blinded, phase I, multicenter trial randomized 36 patients with CCSs to receive either 2.5 mg vericiguat (increased doses every 2 weeks to 5 mg and 10 mg) or placebo. There were 31 patients in the study (21 receiving vericiguat plus nitroglycerin; 10 receiving placebo plus nitroglycerin). The combination of vericiguat with nitroglycerin did not increase the number of adverse events nor the risk of SAEs in patients with CCSs (Boettcher et al., 2022).

#### Praliciguat

Praliciguat (also known as IW-1973) is an sGC stimulator in the clinical stage of testing. It is being studied in clinical studies for the treatment of heart failure and diabetic nephropathy with retained ejection. Treatment with 1–10 mg/kg IW-1973 significantly reduced blood pressure in rats with normal and spontaneous hypertension in nonclinical models. IW-1973 reduced blood pressure, inflammatory factors, and markers of kidney disease, such as proteinuria and renal fibrosis, in rat models. IW-1973 has a wide tissue distribution. It shows renoprotective, anti-inflammatory, and antifibrotic effects (Tobin et al., 2018). In a randomized, placebo-controlled phase I study, different doses of praliciguat were evaluated for safety, tolerability, PK, and pharmacodynamics (PD) in healthy adults (*n* = 44). It was tolerable at various doses, and no SAEs were reported. The most common adverse reactions were headaches and decreased blood pressure. The PK was proportional to the dose, and the effective half-life was 24–37 h. The administration of praliciguat resulted in a rise in plasma cGMP that was proportional to the dosage, indicating activation of sGC. Repeated daily medication can lead to a drop in blood pressure (Hanrahan et al., 2019).

#### Praliciguat in HFpEF

There are no approved sGC stimulants for treating HFpEF, but such treatments are being investigated. CAPACITY HFpEF (NCT03254485) is a phase 2, multicenter, randomized, double-blind, placebo-controlled, parallel-group trial designed to evaluate the safety and efficacy of approximately 181 patients with HFpEF over 12 weeks. A total of 155 participants have completed the trial. The praliciguat group (*n* = 65) showed a change in the peak rate of oxygen consumption (Vo2) of −0.26 mL/kg/min (95% CI, −0.83 to 0.31), compared to 0.04 mL/kg/min (95% CI, −0.49–0.56) in the placebo group (*n* = 78). Changes in the 6MWD were 41.4 m (95% CI, 8.2–74.5) and 58.1 m (95% CI, 26.1–90.1), respectively. No significant benefit of praliciguat was observed in HFpEF compared to placebo over a 12-week follow-up period, but it was accompanied by more hypotension and headache. These findings do not support the use of praliciguat in patients with HFpEF (Udelson et al., 2020).

#### Praliciguat’s different effects in patients with T2D

Praliciguat has renoprotective properties. In a rat model of obese diabetic nephropathy (DN), praliciguat alone reduced proteinuria. Praliciguat monotherapy had no effect on hemodynamics, whereas combined enalapril reduced proteinuria, but monotherapy reduced blood pressure and did not reduce proteinuria (Liu et al., 2020). In a phase II trial (NCT03217591) involving 156 adults with type 2 diabetes, praliciguat did not significantly reduce proteinuria over 12 weeks (Hanrahan et al., 2020b). Furthermore, a mouse model of diet-induced obesity revealed some beneficial metabolic effects of praliciguat (Schwartzkopf et al., 2022). Another phase II, double-blind, placebo-controlled trial of praliciguat (NCT03091920) involved 26 patients with type 2 diabetes combined with hypertension. Praliciguat was well tolerated and showed positive trends in metabolic and BP variables (Hanrahan et al., 2020b).

One study evaluated the effect of praliciguat on hind limb ischemic (HLI) recovery in mice with type 2 diabetes. Praliciguat significantly increased the diameter of their small arteries, decreased the expression of intercellular adhesion molecule 1 (ICAM1), prevented the accumulation of oxidative proangiogenic and proinflammatory muscle fibers, and significantly downregulated the expression of Myh2 and Cxcl12 mRNA in cultured myoblasts (Foussard et al., 2023).

#### Other medicines

##### BAY-747

BAY-747 is a long-acting, next-generation GC stimulator for the treatment of refractory hypertension that is administered once daily and has sustained effects on blood pressure and heart rate (up to 24 h). BAY-747 is taken orally in a single dose of 0.5–15 mg in the form of a polyethylene glycol (PEG) solution and is well tolerated in healthy volunteers. Blood concentrations of BAY-747 peaked within 2–6 h, independent of dose intensity. A single dose of 3.5 mg oral BAY-747 significantly increased heart rate and reduced blood pressure and mean arterial pressure, these effects being most pronounced within the first 4 h after taking the study drug. A single oral dose of 10 mg BAY-747 had significant effects on heart rate, cardiac output, and cardiac index, with maximum effects achieved within 4 h of administration. In the 0.5–20 mg dose range, a single oral dose of BAY-747 did not appear to affect stroke volume (Vakalopoulos et al., 2023).

##### Olinciguat

Olinciguat is a new oral sGC stimulator currently in phase II clinical development (NCT02931565 and NCT03285178). Olinciguat has cardioprotective effects and reduces blood pressure. In addition, it has renal-protective effects, and in a rat ZSF1 model, there is a correlation between decreased levels of glucose, cholesterol, and triglycerides (Zimmer et al., 2020). In a mouse model of TNFα-induced inflammation, olinciguat treatment was associated with reduced levels of soluble adhesion derived from endothelial cells and white blood cells (Tchernychev et al., 2021). Accordingly, it may be suitable for treating diseases characterized by vascular and extravascular lesions as well as a wide range of potential therapeutic applications.

##### MK-2947

MK-2947 is a novel, potent, selective sGC stimulator (Brockunier et al., 2020). A pharmacological study demonstrated that MK-2947 effectively ameliorates angiogenic performance and blunts the myofibroblast-like profibrotic phenotype of SSc dermal microvascular endothelial cells (SSc-MVECs), thus providing new evidence for the benefit of repurposing sGC stimulators for SSc (Romano et al., 2023).

##### CYR715

CYR715, first described by Rennie et al., is a novel carboxylic acid-containing sGC stimulator that exhibited similar dose-dependent hemopharmacology in normotensive rats. Compared to the previously described IWP-051, CYR715 had a better pharmacokinetic profile in rats and exhibits similar dose-dependent hemodynamics in normotensive rats (Rennie et al., 2021). A recent study found that preincubating red blood cells from type 2 diabetes (T2D) patients with CYR715 and administering them to isolated rat hearts enhanced left ventricular diastolic pressure recovery, reduced infarct size, and mitigated endothelial dysfunction. Therefore, CYR715 appears to be an attractive therapeutic strategy for preventing cardiovascular injury in patients with T2D (Jiao et al., 2023).

6MWD, 6-min walking distance; PAH, pulmonary arterial hypertension; CTEPH, chronic thromboembolic pulmonary hypertension; PVR, pulmonary Vascular resistance; IIP, idiopathic interstitial pneumonias; dcSSc, diffuse cutaneous systemic sclerosis; AEs, adverse events; SAEs, serious adverse events; mRSS, modified Rodnan skin score; HF, heart failure; NT-proBNP, N-terminal pro-B-type natriuretic peptide; HF, heart failure; LVEF, left ventricular ejection fraction; LVEDV, left ventricular end-diastolic volume; LVESV, left ventricular end-systolic volume; LAV, left atrial volume; KCCQ, Kansas City Cardiomyopathy Questionnaire; CCSs, chronic coronary syndromes; TEAEs, treatment-emergent adverse events; HFpEF, heart failure with preserved ejection fraction; ABPM, ambulatory BP monitoring; HOMA-IR, homeostatic model assessment of insulin resistance; SAPH, sarcoidosis associated pulmonary hypertension; ADEs, adverse events resulting in study drug discontinuation; BFT, supine bolus flow time.

## Soluble guanylate cyclase activators

While sGC stimulators target reduced and heme-containing forms of sGC, sGC activators target oxidized or heme-free sGC. Since the status of these enzymes occurs mainly in the condition of diseases accompanied by oxidative stress, this binding pattern is quite attractive for the clinical use of activator drugs (Evgenov et al., 2006). Cinaciguat, a type of amino dicarboxylic acid, is the first characteristic drug of this new sGC activator class (Stasch et al., 2002). Although other sGC activators have been identified, no sGC activators are available to patients.

### Cinaciguat

A 1997 ultrahigh-throughput screening (uHTS) identified cinaciguat as an sGC activator (Sandner et al., 2021b). As a direct, NO-independent activator of sGC, cGMP levels directly increase in the presence of heightened oxidative stress and impaired endothelial function, which could yield notable efficacy. However, it increases the risk of low blood pressure (Mitrovic et al., 2011).

### Cinaciguat in PAH

In a randomized, double-blind, multicenter, multinational phase IIb study (NCT00559650), cinaciguat significantly reduced pulmonary capillary wedge pressure (PCWP) and mean right atrial pressure in patients with decompensated chronic congestive heart failure. There was also a decrease in both pulmonary and systemic vascular resistance, as well as a reduction in mean arterial pressure, and the cardiac index increased (Mitrovic et al., 2011). Cinaciguat was associated with 71% of adverse events, and placebo was associated with 45%. There were no adverse events associated with 30-day mortality. When the dose was increased to 200 g/h, hypotensive events increased, and the trial was terminated (Erdmann et al., 2013).

### Cinaciguat in acute HF

Three phase IIb trials, including COMPOSE 1 (NCT01065077), COMPOSE 2 (NCT01067859), and the COMPOSE Early Trial (NCT01064037), were subsequently conducted to investigate the safety and efficacy of varying doses of cinaciguat 200 μg/h *versus* placebo in treating patients with acute HF initiated at different time points. However, because hypotensive events occurred and no significant benefit was observed, the clinical development of cinaciguat was discontinued (Breitenstein et al., 2017). The clinical trials led to the discontinuation of cinaciguat. Most of the research since then has been conducted on animals (Benaldo et al., 2022; Dai and Stuehr, 2023).

### Other medicines

#### Ataciguat

Ataciguat, formerly known as HMR 1766, is an anthranilic acid derivative that is a novel sGC activator (Schafer et al., 2010). Researchers found that ataciguat normalized vasodilation and the vascular response to exogenous NO in rats with congestive heart failure and reduced platelet activation (Schafer et al., 2010). In a rat model of inflammatory chronic renal impairment, ataciguat exhibited beneficial BP-independent effects on kidney structure and urinary albumin excretion (Benz et al., 2007). Ataciguat is currently being studied in clinical trials for numerous indications, including changes in tolerated blood pressure and orthostatic tolerance (i.e., ability to stand without passing out) in patients with mild to moderate calcified aortic stenosis (NCT02049203), effectiveness in relieving patients with neuropathic pain (NCT00799656), improving claudication in patients with PAD (NCT00443287), and slowing the progression of valve calcification in patients with moderate calcific aortic valve stenosis (NCT02481258). However, the results of these clinical trials have not yet been made publicly available.

#### MGV354

MGV354 is a novel sGC activator that effectively lowers IOP in glaucoma models in preclinical studies (Prasanna et al., 2018). Unfortunately, in a clinical trial (NCT02743780), MGV354 did not cause a statistically significant reduction in IOP compared to placebo (Stacy et al., 2018).

#### Mosliciguat

Mosliciguat (BAY 1237592) is an sGC activator designed for topical application in the lung for the treatment of PAH. Inhalation of mosliciguat specifically activates apo-sGC, leading to a selective effect in the lung (Becker-Pelster et al., 2022). Mosliciguat was shown to activate heme-free NO-GC and improve cardiopulmonary circulation in minipigs and rats (Becker-Pelster et al., 2022). Based on these results, mosliciguat is currently in phase Ib clinical development (NCT03754660) as an inhaled therapy for PAH.

#### Runcaciguat

As a once-daily oral sGC activator, runcaciguat demonstrated good PK distribution when administered by Bayer (Hahn et al., 2021). Preclinical studies have demonstrated that runcaciguat may be effective in preventing CKD caused by hypertension, diabetes, and obesity (Benardeau et al., 2021). The oral sGC activator runcaciguat is currently in a phase II clinical program for patients with proteinuric CKD (NCT04507061).

#### BI 685509

BI 685509 is an orally bioavailable, potent sGC activator that exhibits significant renal protection properties and antifibrotic activity in preclinical models of kidney disease and injury (Reinhart et al., 2023). In addition, in a preclinical rat model of thioacetamide-induced nodularity, hepatic fibrosis, portal hypertension and portosystemic shunts, BI 685509 reduced Sirius red morphometry (SRM) by 38%, alpha-smooth muscle actin (αSMA)-positive area by 55%, portal pressure by 26% and portal shunt by 10%; hence, it could be used as a potential treatment for cirrhosis-associated portal hypertension (Jones et al., 2023). BI 685509 effectively inhibited the induction effect of activated platelet-rich plasma on the SSC-related chemokine CXCL4, more strongly than riociguat did (Nabozny et al., 2023). These findings suggest that BI 685509 is a new drug to treat SSc that is superior to riociguat. In a phase Ib study (NCT03165227), BI 685509 was generally well tolerated in patients with diabetic kidney disease (DKD) (Cherney et al., 2023). BI 685509 is currently in phase II trials for CKD (NCT04736628) and DKD (NCT04750577). Studies are currently underway on the indications of PAH and CTEPH (NCT03754660) and SSc (NCT05559580).

#### BI 703704 and GSK2181236A

The sGC activators BI 703704 and GSK2181236A have demonstrated sustained protection against preclinical models of CKD (Costell et al., 2012; Stasch et al., 2015; Hu et al., 2022). The results of these preclinical studies need to be confirmed in humans before these agents can be considered alternatives to current recommended treatments.

## Guanylate cyclase-C agonists

GC-C is a transmembrane protein receptor that has received increasing attention for its importance in digestive diseases. It plays a key role in regulating water and electrolyte balance, maintaining gastrointestinal function, relieving abdominal pain, controlling inflammation, regulating intestinal ecology, inhibiting tumor growth and regulating cell proliferation and is considered a potential therapeutic target for digestive system diseases (Waldman and Camilleri, 2018). It was discovered in 1970 as a receptor for heat-stable endotoxins of exogenous diarrhea-causing bacteria and can be detected not only in intestinal mucosal cells but also in primary and metastatic colorectal cancer, peripheral blood, lymph nodes and liver tissues (Smith and Gyles, 1970). The signaling pathway of GC-C/cGMP has a significant impact on digestive disorders, and agonists are on the market to treat these associated gastrointestinal conditions. GC-C agonists include natural and synthetic ligands; natural ligands include endogenous ligands such as uroguanylin and guanylin, exogenous ligands such as heat-resistant enterotoxin, and synthetic ligands such as linaclotide, plecanatide and dolcanatide (Kuhn, 2016). The current progress of GC-C agonists is summarized in Table 3.

**TABLE 3:** The current progress on guanylate cyclase-C agonists.

Drug	Trial name	Design	Population	Dose	Trial ID	Endpoint	Safety outcome	Stage of development	Conclusion	Reference
Linaclotide	/	Phase III, International, Multicenter, Randomized, Double-blind, Placebo-controlled, Parallel-group Trial	Patients with IBS-C	290 µg daily	NCT01880424	Composite Endpoint of Abdominal Pain and IBS	AEs	Completed	Linaclotide was efficacious and well-tolerated in Chinese	Liang and Liang (2023)
									Patients with IBS-C, with rapid onset of effect	
Linaclotide	/	Phase IIIb, Randomized, Double-blind, Placebo-controlled, Parallel-group Trial	Patients with IBS-C	290 µg daily	NCT03573908	Change in abdominal Score	TEAEs	Completed	Linaclotide significantly reduced multiple abdominal symptoms important to patients with IBS-C	Brenner et al. (2023)
Linaclotide	/	Phase II Multicenter, Randomized, Double-Blind, Placebo-Controlled, Parallel Group Trial	Patients with OIC	145 µg or 290 µg daily	NCT02270983	change in SBMs/week	AEs and SAEs	Completed	Linaclotide significantly improved OIC symptoms and was well tolerated in patients with chronic noncancer pain	Brenner et al. (2020)
Linaclotide	/	Phase I, Randomized, Placebo-Controlled Trial	Patients with Colorectal Cancer	0.87 mg daily	NCT01950403	difference in mean cGMP levels after 7 days	AEs	Completed	Linaclotide was associated with homeostatic signaling, including phosphorylation of vasodilator-stimulated phosphoprotein and inhibition of proliferation quantified by fewer Ki67-positive epithelial cells	Weinberg et al. (2017)
Linaclotide	/	Phase III Randomized, Double-blind, Placebo-controlled trial	Patients with CC	0.5 mg daily	NCT02809105	Change from baseline in average weekly SBM frequency	AEs 、SAEs	Completed	Linaclotide 0.5 mg/day is effective and safe in Japanese CC patients	Fukudo et al. (2019)
Plecanatide	The CIC3 Study	Randomized, Double-Blind, Placebo-Controlled trial	Patients with CIC	3 mg or 6 mg daily	NCT01982240	percentage of CSBM	AEs	Completed	Plecanatide significantly improved constipation and related symptoms with a low rate of adverse events	Miner et al. (2017)
Plecanatide	The National CIC3 Study	Randomized, 12-Week, Double-Blind, Placebo-Controlled trial	Patients with CIC	3 mg or 6 mg daily	NCT02122471	Number of Durable Overall CSBM Responders	AEs 、SAEs and TEAEs	Completed	Plecanatide has a positive efficacy and safety profile in CIC patients	DeMicco et al. (2017)

IBS-C, irritable bowel syndrome with constipation; AEs, adverse events; SAEs, serious adverse events; TEAEs, treatment-emergent adverse events; OIC, opioid-induced constipation; SBMs, spontaneous bowel movements; CIC, chronic idiopathic constipation; CSBM, complete spontaneous bowel movement; CC, chronic constipation.

### The GC-C/cGMP signaling pathway

**FIGURE 2:**
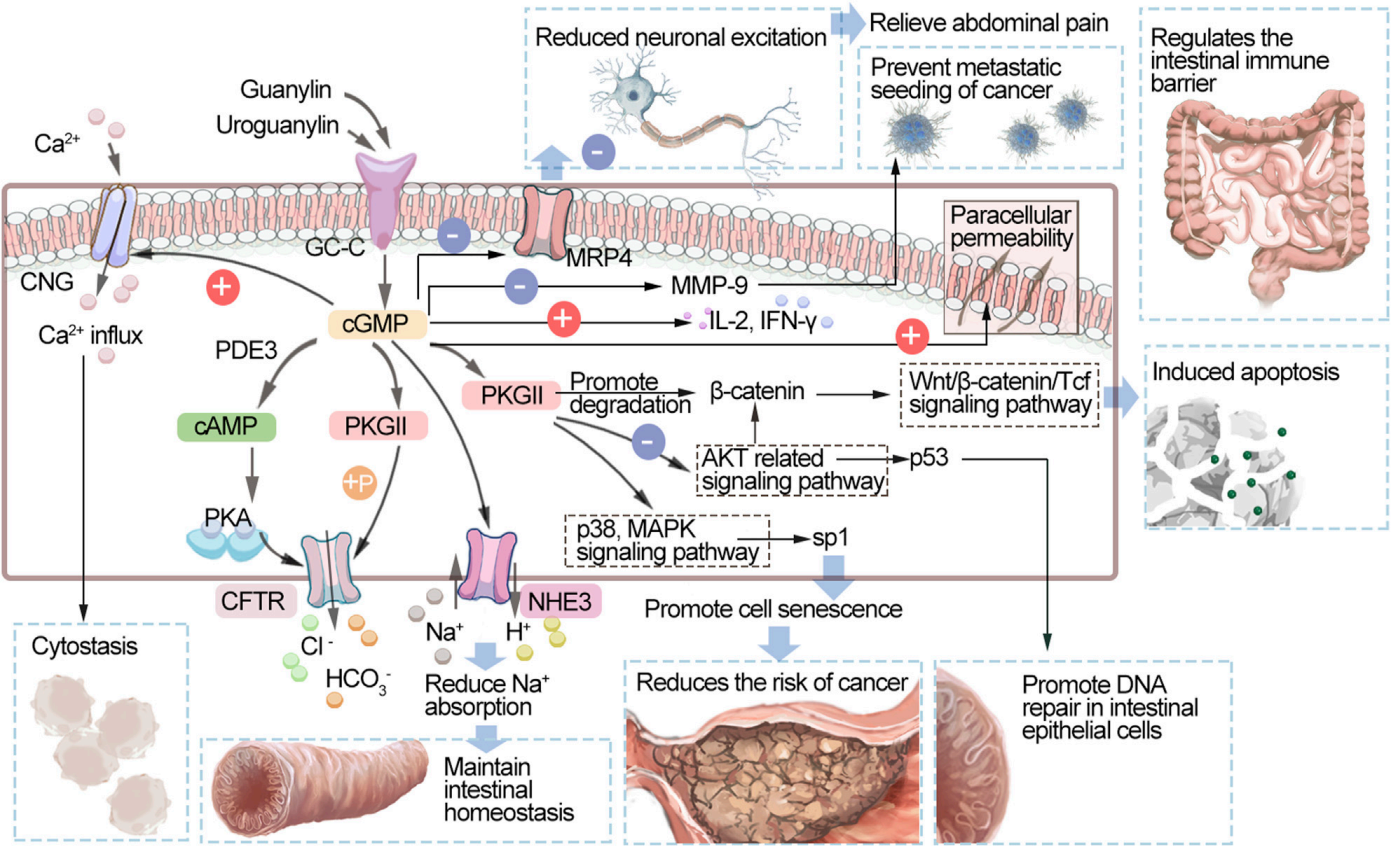
Schematic diagram of the GC-C/cGMP signaling pathway. GC-C, guanylate cyclase C; cGMP, cyclic guanosine monophosphate; PDEs, phosphodiesterases; cAMP, cyclic adenosine monophosphate; PKA, protein kinase A; PKG, protein kinase; CNGs, cyclic nucleotide-gated ion channels; CFTR, cystic fibrosis transmembrane regulator; NHE3, Na^+^/H^+^ exchanger 3; MRP4, multidrug resistance-associated protein 4; Akt, protein kinase B; p38 MAPK, p38 mitogen-activated protein kinase; MMP-9, matrix metalloproteinase-9; IL-2, interleukin 2; IFN-γ, Interferon γ.

As shown in Figure 2, intestinal guanylin and uroguanylin are effective regulators of fluid ion homeostasis. They are secreted by various cells of the intestinal mucosa, including intestinal chromaffin cells, epithelial cells, goblet cells, and Pan’s cells. These peptide hormones act as receptor GC-C ligands that produce intracellular cGMP and activate PKGII. PKGII phosphorylates the cystic fibrosis transmembrane conduction regulator (CFTR) and increases the secretion of chloride ions (CI^−^) into the intestinal lumen. cGMP can also increase the content of cyclic adenosine monophosphate (cAMP) by inhibiting the activity of PDE3. cAMP activates PKA and coactivates CFTR along with PKGII. Promoting the discharge of chloride ions and bicarbonate (Vaandrager, 2002), cGMP activates HCO_3_^−^ secretion through an unknown mechanism. In addition, cGMP can inhibit the sodium/hydrogen exchanger NHE3, thereby reducing the absorption of sodium, preventing hypernatremia and unnecessary hypervolemic shock, and maintaining the fluid balance in the intestine. By these mechanisms, cGMP can maintain the hydration state of colon mucus and ion homeostasis (Brierley, 2012; Waldman and Camilleri, 2018; Samanta and Chaudhuri, 2021). In addition, it can regulate the intestinal immune barrier and increase the levels of IL-2 and IFN-γ and paracellular permeability (Xing et al., 2021). Abdominal pain is a major symptom of inflammatory bowel disease (IBD), and therapies modulating the GC-C/cGMP pathway promote visceral analgesia in patients with these diseases in animal models and clinical trials (Waldman and Camilleri, 2018). The mechanism of its analgesia is mainly through the afferent pathway involved in the regulation of gastrointestinal pain. After receptor–ligand binding in epithelial cells, elevated intracellular cGMP can be transported to the extracellular space through the silencing of the multidrug resistance-related protein 4 cyclic nucleoglycine efflux pump located on the basolateral membrane, reducing the excitation of submucosal afferent neurons and relieving abdominal pain. Another mechanism underlying this action is the prevention of intraluminal factors from affecting pain afferents and immune mechanisms in the lamina propria and other areas to indirectly promote analgesia (Shailubhai et al., 2015; Brierley et al., 2022). It can also inhibit the proliferation of intestinal epithelial cells and maintain genomic stability, thus inhibiting the occurrence of intestinal tumors. Following the activation of GC-C, the level of cGMP increases to activate cyclic phospho-dependent PKGII, which can inhibit the protein kinase B (Akt)-related signaling pathway and thus upregulate the expression of the tumor suppressor p53. p38 MAPK is also activated, which increases the phosphorylation of the transcription factor Sp1, leading to the accumulation of p21 in cells, which promotes cell aging and reduces the risk of cancer (Basu et al., 2014). Activation of GC-C induces apoptosis by promoting the degradation of β-catenin and opposing the pro-proliferative Wnt/β-catenin/Tcf-4 signaling pathway (Thompson et al., 2000). Because Akt enhances β-catenin activity either directly or indirectly, the suppression of Akt signaling may also be associated with the reduction of β-catenin/T-cell factor (TCF) transcriptional activity mediated by GC-C (Fang et al., 2007). As the GMP concentration increases, cyclic nucleotide-gated ion channels (CNGs) are activated, Ca^2+^ inflow is promoted, and cytostatic activity is mediated (Kazerounian et al., 2005). GC-C signaling can also block metastasis by inhibiting matrix metalloproteinase-9 (MMP-9), which is produced by colorectal cancer cells (Lubbe et al., 2009). PKGII can also promote DNA repair in intestinal epithelial cells, thereby protecting intestinal epithelial tight junction proteins, maintaining intestinal wall integrity and reducing the risk of tumor development (Rappaport and Waldman, 2020; Loretah et al., 2021).

#### Linaclotide

Linaclotide, a 14-amino acid peptide, was approved by the FDA in 2012 as an oral drug for the treatment of chronic constipation (CC) and constipation-type irritable bowel syndrome (IBS-C). It acts on the GC-C receptor on the luminal membrane, resulting in increased intracellular phosphorylation of cGMP and pVASpser239. The mechanisms underlying its effects include increased bicarbonate secretion during CFTR expression or functional loss, increased chloride and bicarbonate secretion into the intestine, inhibited sodium ion absorption, and thus increased water secretion into the lumen and improved defecation (Love et al., 2014; Ahsan et al., 2017; Sarthi et al., 2023). The pharmacological activity of linaclotide is limited to the gastrointestinal tract, and its oral bioavailability is low, so systemic side effects are unlikely. After oral linaclotide is taken, approximately 3%–5% of the active peptide is excreted in the stool. Linaclotide and its metabolites are excreted through the kidneys (33%–45%) and biliary tract (48%–59%). Its dosage should be adjusted with particular care in patients with moderate liver impairment or mild to severe liver impairment (data are not available for patients with severe liver impairment). It is metabolized by multiple cytochrome P450 (CYP) enzymes and has no effect on major CYP subtypes, giving it a low risk of clinically relevant drug interactions (Frey et al., 2018). A therapeutic dose of linaclotide should be taken at least 30 min before meals, as effectiveness and tolerability can be affected by high-fat foods (Bassotti et al., 2018).

#### Linaclotide in chronic constipation syndrome

Linaclotide was evaluated at doses between 62.5 g and 600 g for efficacy and safety in patients (*n* = 4,107) with CC in a meta-analysis. More patients completed spontaneous defecation (CSBM) at different doses, which was more significant in the very low-dose group, and there were no adverse reactions in the high-dose group (Yang and Lei, 2021). Japanese researchers (NCT02809105) found that linaclotide at a dose of 0.5 mg/day was effective and safe in patients with CC, with mild and occasional adverse reactions being the most common (Fukudo et al., 2019). A prospective study from China evaluated patients taking linaclotide (*n* = 97) on bowel movements, abdominal symptoms, IBS Symptom Severity Scale (IBS-SSS), and IBS Quality of Life Questionnaire (IBS-QOL) and found a significant increase in weekly bowel movements and a significant improvement in patients’ quality of life. Diarrhea occurred in 11 cases (11.3%). IBS-C symptoms and severity were improved with linaclotide, and the drug was safe and effective (Liu et al., 2022). Another phase III clinical trial involving 659 Chinese IBS-C patients also showed that linaclotide (290 µg/day) was effective and well tolerated in Chinese IBS-C patients with rapid onset of action (NCT01880424) (Peng et al., 2022). Another study found that linaclotide reduced reaction time in patients with IBS-C (Brenner et al., 2023). In a trial in the elderly population, the most common side effect was diarrhea, but the incidence of diarrhea and constipation in elderly patients, even at reduced doses, did not differ significantly from that in nonelderly patients, and multivariate analysis showed that age, sex, and dose were not associated with diarrhea caused by linaclotide treatment. Thus, linaclotide was effective and safe in elderly patients (Chang et al., 2021; Ishigo et al., 2021). In special pediatric populations, AEs are relatively common, although studies have found that nearly half of children with FC or IBS-C benefit from linaclotide treatment, so further research is needed (Baaleman et al., 2021). In a cohort study of patients with SSc, 31 patients were treated with linaclotide. Twenty-eight of the 31 patients responded to treatment, while only three (9.7%) reported ineffective or intolerable side effects. Diarrhea, cramping, and bloating were the most commonly reported side effects (11/31, 35%). Linaclotide is a well-tolerated, effective drug that can be used to treat refractory symptoms of a low GI score on SSc (Dein et al., 2021). A multicenter phase II clinical study evaluated the efficacy and safety of linaclotide in the treatment of opioid-induced constipation (OIC) in patients with chronic noncancer pain syndrome (NCT02270983). Compared with placebo, linaclotide significantly improved stool consistency, diarrhea, bloating, and treatment satisfaction scores (*p* < 0.05). Linaclotide significantly improved OIC symptoms and was well tolerated in patients with chronic noncancerous pain (Brenner et al., 2020).

#### Linaclotide in visceral pain and colon cancer

Linaclotide treatment was found to reduce vaginal hyperalgesia and mechanical hyperalgesia associated with endometriosis through viscerovaginal crosstalk (Ge et al., 2019). In patients with colon cancer, a US phase I clinical trial (NCT01950403) showed that administration of linaclotide (870 μg/d) for 7 days after oral preparation of the intestine with polyethylene glycol increased the level of cGMP and reduced the proportion of Ki-67-positive colon epithelial cells (a higher proportion of Ki-67-positive cells suggested a faster rate of cell proliferation). These results suggest that linaclotide inhibits colonic epithelial cell proliferation in human colons (Weinberg et al., 2017).

#### Plecanatide

An oral GC-C agonist, plecanatide, consists of a 16-amino acid synthetic peptide equivalent to human uroguanylin and is used to treat gastrointestinal (GI) disorders. For the treatment of chronic idiopathic constipation (CIC) in adults, plecanatide received its first global approval in 2017 (Al-Salama and Syed, 2017; Rao, 2018). Plecanatide metabolism occurs in the gastrointestinal tract. After oral administration of 3 mg of plecanatide, blood levels of plecanatide and its active metabolites were lower than detectable levels. Standard pharmacokinetic parameters cannot be calculated, and the amount of plecanatide or its metabolites in the tissue is negligible due to the small amount of drug absorbed (Miner, 2020).

In two large randomized, double-blind, placebo-controlled studies evaluating the efficacy and safety of plecanatide (3 mg, 6 mg) vs placebo in patients with CIC, plecanatide treatment also significantly reduced the severity of other CIC symptoms (tension, consistent stools, bloating). In addition, the satisfaction and quality of life of patients treated with plecanatide improved significantly. The low incidence of adverse reactions after plecanatide treatment shows that it is safe and effective for CIC. In addition, plecanatide combined with acid suppressants is safe and effective in patients with CIC (DeMicco et al., 2017; Miner et al., 2017).

A meta-analysis evaluating the efficacy and tolerability of GC-C agonists included eight randomized controlled trials with 10,369 patients. Both drugs were effective in treating CIC, and the incidence of diarrhea was higher than that in the placebo group. Linaclotide and plecanatide were similar in efficacy and tolerability to IBS-C and CIC. There was no difference in the incidence of adverse reactions (diarrhea) between linaclotide and plecanatide (Shah et al., 2018).

#### Other medicines

##### Dolcanatide

Dolcanatide (SP-333), an oral uroguanylinoid, is replaced by selected D-amino acids to enhance stability and extend persistence, activating GUCY2C in the small and large intestines. A phase I double-blind, placebo-controlled trial (NCT03300570) of 27 mg dolcanatide administered orally daily for 7 days in healthy volunteers did not show activation of GUCY2C in distal rectal epithelial cells, as quantified by the accumulation of its product cGMP. These data suggest that the high stability of dolcanatide and its persistence along the rostral–caudal axis of the small and large intestines are insufficient to regulate GUCY2C throughout the colorectal region to prevent tumorigenesis. These results highlight the importance of developing GUCY2C anticancer agonists that target colorectal release and activity (Weinberg et al., 2021). Current research on dolcanatide focuses on its potential use for the treatment of colon cancer.

## Summary

GC is widely distributed throughout the human body, mostly as sGC and GC-C, which catalyze GTP and thus increase the intracellular content of the second messenger cGMP, leading to the modulation of various intracellular physiological regulatory processes. The development of drugs on the basis of functional modulation of the GC pathway has been a fast-moving area of research over the past few years. A series of clinical studies have validated the therapeutic potential of sGC stimulators in patients with HF and PAH. Due to promising clinical trial results, riociguat and vericiguat have been approved by the US FDA for the treatment of chronic heart failure and pulmonary hypertension. In addition, the role of sGC stimulants in improving cGMP signaling might enable them to play an active role in a wide range of clinical indications, such as metabolic diseases, fibrotic diseases, urinary diseases, and neurodegenerative diseases. However, clinical studies have shown that the benefit for dcSSc patients and patients with diabetic nephropathy is debatable, and more clinical studies are needed to support its use or nonuse in them. In contrast to sGC stimulants, the treatment potency of sGC activators is not fully clarified, although they can bind to sGC when the body is in a disease condition involving oxidative stress. The development of this class of drugs is still at the clinical study stage. In intestinal epithelial cells, GC-C is the dominant supplier of cGMP. The GC-C molecule is important for maintaining fluid and ion homeostasis in the intestinal tract, and linaclotide and plecanatide are the main drugs targeting it. Preclinical and clinical evidence shows that modulation of GC-C may improve symptoms and be tolerated by patients with IBS-C and CIC. Recent studies have shown that GC-C is also relevant to intestinal inflammation, dysbiosis and cancer, the specific mechanisms of which remain to be explored. In conclusion, cGMP has a broad spectrum of physiological effects, which gives it considerable development prospects. We systematically reviewed the transduction procedures of the NO-sGC-cGMP signaling pathway and the potential use of sGC stimulators and GC-C stimulators. Novel compounds are being developed based on the structures of sGC and GC-C, some of which are being studied in clinical trials. These clinical results may open up new therapeutic approaches for cardiovascular, renal and other diseases. Various questions remain regarding the mechanisms of their effects and the therapeutic potential of these treatments for diseases besides PAH and HF, and much more research is needed in the future.

## Figures and Tables

**Table:** 

**GTP**	guanosine triphosphate
**cGMP**	cyclic guanosine monophosphate
**sGC**	soluble guanylate cyclase
**NO**	nitric oxide
**CO**	carbon monoxide
**H-NOX**	N-hemoglobin-nitric oxide
**GC-C**	guanylate cyclase C
**ECD**	extracellular domain
**NOS**	nitric oxide synthases
**eNOS**	endothelial nitric oxide synthase
**nNOS**	neuronal nitric oxide synthase
**PKGs**	protein kinases
**PDEs**	phosphodiesterases
**PLB**	phospholamban
**RyR2**	Cardiac ryanodine receptor
**SERCA**	sarco endoplasmic reticulum Ca2+-ATPase
**ER**	endoplasmic reticulum
**LTCCs**	L-type calcium channels
**TRPC6**	transient receptor potential canonical channel type 6
**TnI**	troponin I
**TTN**	titin
**CMyBP-C**	cardiac myosin-binding protein-C
**VSMC**	venous smooth muscle cell
**MLCP**	myosin light chain phosphatase
**RhoA**	Ras homolog family member A
**RGS-2**	regulator of G-protein signaling 2
**IP3**	inositol 1,4,5-trisphosphate
**Rap1Gap2**	Rap1 GTPase-activating protein 2
**Rap1b**	Ras-related protein 1
**IRAG**	IP3-induced calcium release
**GRP2**	guanyl-releasing protein 2
**BKCa**	calcium-sensitive potassium channels
**PASMCs**	pulmonary artery smooth muscle cells
**MLCK**	myosin light chain kinase
**VASP**	vasodilator-stimulated phosphoprotein
**BMP**	bone morphogenetic protein
**SMAD**	small mothers against decapentaplegic
**PDE5**	phosphodiesterase 5
**NFAT**	calcineurin-nuclear factor of activated T cells
**HF**	heart failure
**CVD**	cardiovascular disease
**HFrEF**	heart failure with reduced ejection fraction
**HFpEF**	heart failure with preserved ejection fraction
**ERK**	extracellular signal-regulated kinase
**ECM**	extracellular matrix
**CKD**	chronic kidney disease
**PAH**	pulmonary arterial hypertension
**CTEPH**	chronic thromboembolic pulmonary hypertension
**AE**	adverse event
**SAE**	serious adverse event
**RV**	right ventricular
**PH-IIP**	pulmonary hypertension associated with idiopathic interstitial pneumonia
**6MWD**	6-min walk distance
**PAP**	pulmonary artery pressure
**PVR**	pulmonary vascular resistance
**RAP**	right atrial pressure
**CI**	cardiac index
**CO**	cardiac output
**BPA**	balloon pulmonary angioplasty
**SAPH**	sarcoidosis associated pulmonary hypertension
**PK**	pharmacokinetics
**WHO-FC**	World Health Organization Functional Classification
**TAC**	transverse aortic constriction
**LVEF**	left ventricular rejection fraction
**LVM/BW**	left ventricular mass to body weight ratio
**MYH7**	myosin heavy chain 7
**PLN**	cardiac phosphoprotein
**ANKRD1**	ankyrin repeat domain-containing protein 1
**dcSSc**	diffuse cutaneous systemic sclerosis
**mRSS**	modified Rodnan skin score
**HUVECs**	human umbilical vein endothelial cells
**PACs**	pro-angiogenic cells
**CCSs**	chronic coronary syndromes
**BP**	blood pressure
**SBP**	systolic blood pressure
**DBP**	diastolic blood pressure
**PD**	pharmacodynamics
**ECG**	electrocardiogram
**DN**	diabetic nephropathy
**RPTC**	renal proximal tubular epithelial cells
**T2D**	type 2 diabetes
**RHI**	reactive hyperemia index
**HLI**	hind limb ischemia
**ICAM1**	intercellular adhesion molecule 1
**Myh2**	MyHCIIa
**Cxcl12**	C-X-C motif chemokine ligand 12
**PEG**	polyethylene glycol
**SSc-MVECs**	SSc dermal microvascular endothelial cells
**RBCs**	red blood cells
**TGFβ**	transforming growth factors β
**CYP450**	cytochrome P450
**cGKI**	cGMP-dependent protein kinase type I
**CLb**	blood clearance
**uHTS**	ultrahigh-throughput screening
**PCWP**	pulmonary capillary wedge pressure
**ADHF**	acute decompensated heart failure
**IOP**	intraocular pressure
**CAVS**	calcific aortic valve stenosis
**SMA**	smooth muscle actin
**PRP**	platelet rich plasma
**SRM**	sirius red morphometry
**DKD**	diabetic kidney disease
**CFTR**	cystic fibrosis transmembrane regulator
**CI-**	chloride ions
**cAMP**	cyclic adenosine monophosphate
**PKA**	protein kinase A
**HCO3-**	bicarbonate ion
**NHE3**	Na+/H+ exchanger 3
**IBDs**	inflammatory bowel diseases
**MRP4**	multidrug resistance-associated protein 4
**Akt**	protein kinase B
**p38 MAPK**	p38 mitogen-activated protein kinase
**CNGs**	cyclic nucleotide-gated ion channels
**MMP-9**	matrix metalloproteinase-9
**IBS-C**	irritable bowel syndrome with constipation
**CC**	chronic constipation
**CSBM**	complete spontaneous defecation
**IBS-SSS**	IBS Symptom Severity Scale
**IBS-QOL**	IBS Quality of Life Questionnaire
**CI**	confidence interval
**FC**	functional constipation
**GI**	gastrointestinal
**OIC**	opioid-induced constipation
**SBMs**	spontaneous bowel movements
**CIC**	chronic idiopathic constipation
**AUC**	area under the concentration-time curve
**Cmax**	maximum concentration
**T1/2**	drug half-life
**GUCY2C**	Guanylate cyclase C
**TEAEs**	treatment-emergent adverse events
**IIP**	Idiopathic Interstitial Pneumonias
**NT-proBNP**	N-terminal pro-B-type natriuretic peptide
**LVEDV**	left ventricular end-diastolic volume
**LVESV**	left ventricular end-systolic volume
**ABPM**	Ambulatory BP monitoring
**HOMA-IR**	Homeostatic Model Assessment of Insulin Resistance
**ADOs**	adverse events resulting in study drug discontinuation
**BFT**	Supine Bolus Flow Time
**VAS**	visual analog scale
**LOCF**	last observation carried forward
**NPSI**	Neuropathic Pain Symptom Inventory
**PAD**	Peripheral Arterial Disease
**ICD**	initial claudication distance
**AVC**	Aortic Valve Calcification
**UACR**	urinary albumin-to-creatinine ratio
**NPDR**	nonproliferative diabetic Retinopathy
**DRSS**	Diabetic Retinopathy Severity Scale
**CSPH**	clinically significant portal hypertension
**HVPG**	hepatic venous pressure gradient
**CTCAE**	Common Terminology Criteria for Adverse Events
**FVC**	forced vital capacity
**KCCQ-CSS**	Kansas City Cardiomyopathy Questionnaire Clinical Summary Score
**EQ-5D**	5-dimension EuroQol questionnaire
**LAV**	left atrial volume
